# Resveratrol inhibits prostaglandin formation in IL-1β-stimulated SK-N-SH neuronal cells

**DOI:** 10.1186/1742-2094-6-26

**Published:** 2009-09-14

**Authors:** Lena Wendeburg, Antonio Carlos Pinheiro de Oliveira, Harsharan S Bhatia, Eduardo Candelario-Jalil, Bernd L Fiebich

**Affiliations:** 1 Neurochemistry Research Group, Department of Psychiatry, University of Freiburg Medical School, Hauptstrasse 5, D-79104 Freiburg, Germany; 2 VivaCell Biotechnology GmbH, Ferdinand-Porsche-Str. 5, D-79211 Denzlingen, Germany; 3 Department of Neurology, University of New Mexico Health Sciences Center, Albuquerque, NM 87131, USA

## Abstract

Resveratrol, a polyphenol present in grapes and red wine, has been studied due to its vast pharmacological activity. It has been demonstrated that resveratrol inhibits production of inflammatory mediators in different *in vitro *and *in vivo *models. Our group recently demonstrated that resveratrol reduced the production of prostaglandin (PG) E_2 _and 8-isoprostane in rat activated microglia. In a microglial-neuronal coculture, resveratrol reduced neuronal death induced by activated microglia. However, less is known about its direct roles in neurons. In the present study, we investigated the effects of resveratrol on interleukin (IL)-1β stimulated SK-N-SH cells. Resveratrol (0.1-5 μM) did not reduce the expression of cyclooxygenase (COX)-2 and microsomal PGE_2 _synthase-1 (mPGES-1), although it drastically reduced PGE_2 _and PGD_2 _content in IL-1β-stimulated SK-N-SH cells. This effect was due, in part, to a reduction in COX enzymatic activity, mainly COX-2, at lower doses of resveratrol. The production of 8-*iso*-PGF_2α_, a marker of cellular free radical generation, was significantly reduced by resveratrol. The present work provides evidence that resveratrol reduces the formation of prostaglandins in neuroblastoma cells by reducing the enzymatic activity of inducible enzymes, such as COX-2, and not the transcription of the PG synthases, as demonstrated elsewhere.

## Findings

Neuroinflammation is an important component of neurodegenerative diseases and different inflammatory mediators contribute to the process of these disorders. The prostanoids produced from the arachidonic acid (AA) cascade seem to play important roles in these pathological conditions. Among all prostanoids formed from the AA cascade, prostaglandin (PG) E_2 _seems to play an important role in the development of neuroinflammation. In inflammatory conditions, the two enzymes that contribute the most to PGE_2 _production are cyclooxygenase (COX)-2, which converts AA into PGG_2 _and PGH_2_, and microsomal PGE_2 _synthase-1 (mPGES-1), which converts PGH_2 _into PGE_2 _[1]. The involvement of PGE_2_, COX-2 and mPGES-1 in neurodegenerative diseases has been extensively demonstrated [2,3].

Resveratrol (trans-3, 5, 4'-trihydroxystilbene) is a polyphenol found in many plants and in the red wine that reveals several pharmacological actions, including anti-inflammatory properties. Recent reports have evaluated the potential protective role of resveratrol in neurodegenerative conditions. Resveratrol reduces the expression of different inflammatory mediators involved in the progression of neuropathological conditions and it has been shown to provide neuronal protection in different models [4].

Although resveratrol has been shown to reduce the expression of different inflammatory mediators in some cells, including glial cells, its influence in the production of these molecules in neuronal cells has been less studied. Therefore, in the present study, we investigated the effect of resveratrol on the production of prostanoids induced by IL-1β in SK-N-SH cells, a human neuroblastoma cell line.

SK-N-SH cells were obtained from the American Type Culture Collection (HTB-11, Rockville, USA) and were grown in MEM-Earle's medium (PAA, Cölbe, Germany), which does not contain any anti-inflammatory substance. Medium was supplemented with 5% fetal calf serum (PAN, Aidenbach, Germany), 2 mM L-glutamine, 1 mM sodium pyruvate, 40 units/ml penicillin/streptomycin (all purchased from PAA Laboratories, Cölbe, Germany), 0.4% MEM vitamins and 0.4% MEM nonessential amino acids (both purchased from Invitrogen GmbH, Karlsruhe, Germany). Confluent monolayers were passaged routinely by trypsinization. Cultures were grown at 37°C in 5% CO_2 _until 80% confluence, and the medium was changed the day before treatment.

To investigate the effect of resveratrol on the production of inflammatory mediators in neuronal cells, SK-N-SH neuronal cells were pre-incubated with different concentrations (0.001-5 μM) of resveratrol (Sigma-Aldrich, Taufkirchen, Germany) for 30 minutes followed by stimulation with IL-1β (10 U/ml) for 24 h. In control experiments, cells were pre-incubated for 30 min with the following COX inhibitors: SC-560 (5-(4-chlorophenyl)-1-(4-methoxyphenyl)-3-(trifluoromethyl)-1H-pyrazole) (Cayman Chemical Co., Ann Arbor, Michigan, USA), DFU ([5,5-dimethyl-3-(3-fluorophenyl)-4-(4-methylsulphonyl)phenyl-2(5H)-furanone]) and L745,337 ((5-methanesulfonamido-6-(2,4-difluorothiophenyl)-1-indanone)) (both from Merck Frosst, Montreal, Canada). After the 24 h stimulation period, supernatants were harvested for the measurement of the levels of 8-*iso*-PGF_2α _(IUPAC nomenclature: 15-F_2t_-IsoP), PGD_2 _(both from Cayman Chemicals, Ann Arbor, MI, USA), and PGE_2 _(AssayDesign, distributed by Biotrend, Köln, Germany). All measurements were performed according to the manufacturer's instructions. The standards were used in the interval of 3.9-500 pg/ml (detection limit of 5 pg/ml) for 8-*iso*-PGF_2α _and 39-2500 pg/ml (sensitivity of the assay was 36.2 pg/ml) for PGD_2 _and PGE_2_.

The same stimulation was used for Western blot analysis of mPGES-1, COX-1 and COX-2. For mPGES-1, COX-2 and COX-1 immunoblotting, 30 to 50 μg of protein from each sample was subjected to SDS-PAGE (polyacrylamide gel electrophoresis) on a 15% gel under reducing conditions. Primary antibodies were goat anti-COX-2 and anti-COX-1 (M-19 and M-20, respectively, Santa Cruz, Heidelberg, Germany) diluted 1:500 in Tris-buffered saline (TBS) containing 0.1% Tween 20 (Merck, Darmstadt, Germany) and 1% bovine serum albumin (BSA, Sigma), rabbit anti-mPGES-1 (Cayman, 1:500), rabbit anti-actin (Sigma 1:5000).

An AA assay was performed to determine the effect of resveratrol on COX-1 and COX-2 enzymatic activity [5]. To measure total COX activity, SK-N-SH cells were plated in 24-well cell culture plates and pre-incubated with IL-1β (10 U/ml) for 24 h to induce COX-2 protein synthesis. After the pre-incubation, medium was replaced by serum-free medium. Resveratrol was added for 15 min prior to the addition of AA (15 μM final concentration), which was supplemented for another 15 min. In control experiments, cells were pre-incubated for 15 min with the following COX inhibitors: SC-58125 (1-[(4-methysufonyl)phenyl]-3-tri-fluoromethyl-5-(4-fluorophenyl)pyrazole), valeroyl salicylate (VAS) (both compounds obtained from Cayman Chemical Co., Ann Arbor, Michigan, USA) and SC-560. To measure COX-1 activity, the procedure was similar, except that no pre-incubation with IL-1β was performed. Supernatants were collected for determination of PGE_2_. The procedure to measure COX activity by quantification of the PGE_2 _production by enzymatic conversion of AA has been widely used and is well accepted as a method to evaluate potential COX inhibitors [6-10]. Since AA is exogenously added, this assay is largely independent of phospholipase activity.

At least three independent experiments were used for data analysis. All the original data were converted into %-values of IL-1β control and mean ± S.E.M. were calculated. IC_50 _(concentration of the resveratrol that inhibited 50% of the prostaglandin production) was calculated by computerized non-linear regression analysis. Values were compared using one-way ANOVA with *post-hoc *Student-Newman-Keuls test (multiple comparisons).

Resveratrol did not show toxicity at the doses used as evaluated by the ability of the cells to produce ATP (CellTiter-Glo^® ^luminescent cell viability assay kit, Promega, Mannheim, Germany, data not shown).

Considering that resveratrol possesses antioxidant activity [11], we first evaluated whether resveratrol was able to reduce free radical production in IL-1β-stimulated SK-N-SH. Isoprostanes are formed from the arachidonate peroxidation catalysed by free radicals [12] and 8-isoprostanes are accepted as a reliable and sensitive measure of free radical formation [13].

**Figure 1:**
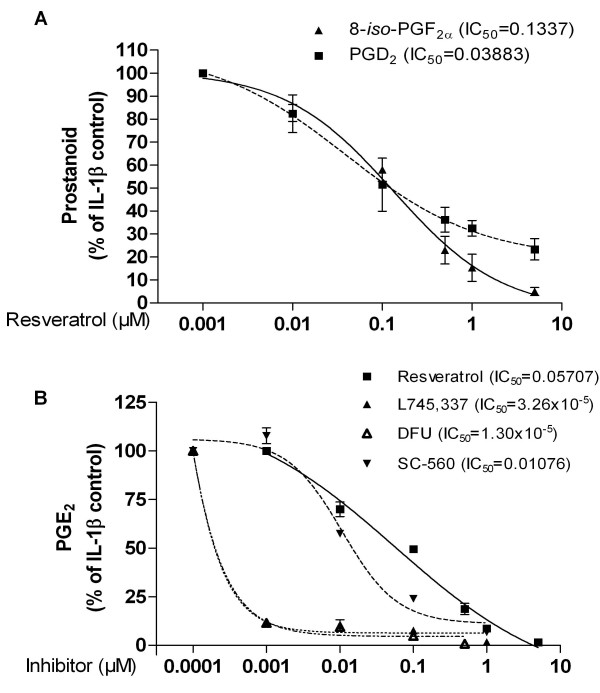
**Resveratrol dose-dependently inhibited IL-1β-induced production of 8-*iso*-PGF_2α_, PGD_2 _(A) and PGE_2 _(B) in SK-N-SH cells**. Selective inhibitors of COX-2 (L745,337 and DFU) and COX-1 (SC-560) were included as controls (**B**). Cells were incubated with the compounds for 30 minutes and subsequently treated with IL-1β (10 U/ml) for 24 h. Data are expressed as mean ± S.E.M. of at least 3 independent experiments.

IL-1β increased the production of 8-*iso*-PGF_2α_, which was strongly reduced by resveratrol (Fig. 1A), even at very low concentrations (IC_50 _= 0.1337 μM), confirming its role as a natural antioxidant.

We have recently shown that resveratrol inhibits the production of PGE_2 _in LPS-stimulated rat microglia [14]. To evaluate whether a similar effect occurs in neuronal cells, we investigated whether resveratrol reduces PGE_2 _production in IL-1β-stimulated SK-N-SH cells. IL-1β (10 U/ml) strongly induced the production of PGE_2 _in neuronal cells. The increased PGE_2 _production at 24 h was drastically reduced by resveratrol, even at very low doses (IC_50 _= 0.05707 μM, Fig. 1B). Moreover, resveratrol also strongly reduced the production of PGD_2 _(IC_50 _= 0.03883 μM, Fig. 1A). To evaluate whether COX inhibitors were able to reduce the production of PGE_2 _in the same conditions, we used COX-1 (SC-560) and COX-2 (L745,337 and DFU) inhibitors. Interestingly, both the COX-1 and the COX-2 inhibitors reduced the production of PGE_2 _in IL-1β-stimulated SK-N-SH cells (Fig. 1B), which indicates that COX-1 activity may also contribute to the production of prostanoids in stimulated cells. Because this experimental set up did not clarify the mechanism of inhibition of the production of prostanoids by resveratrol, we performed further experiments to investigate this issue.

**Figure 2:**
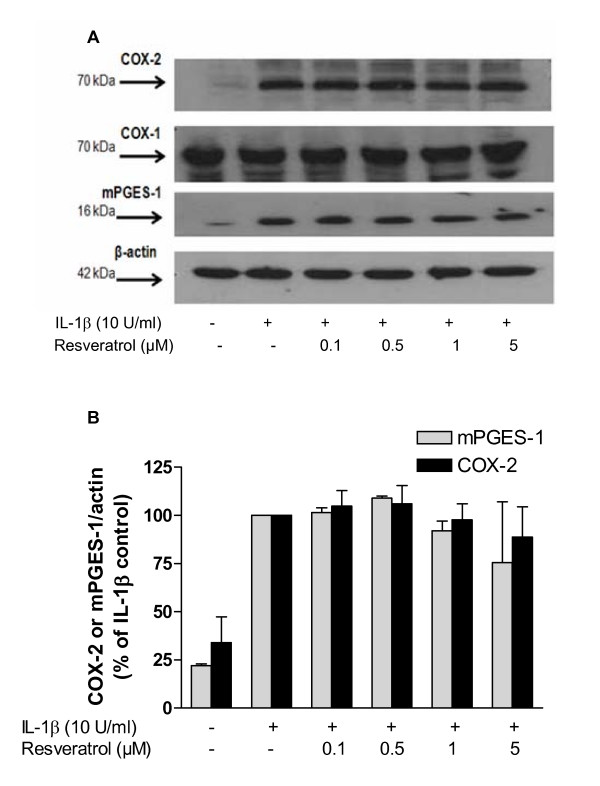
**A and B: Immunoblot analysis of the protein levels of mPGES-1, COX-2 and COX-1 in IL-1β-stimulated SK-N-SH cells treated with resveratrol**. Resveratrol does not inhibit IL-1β-induced levels of mPGES-1, COX-2 in SK-N-SH cells. Cells were incubated with resveratrol for 30 minutes and subsequently treated with IL-1β (10 U/ml) for 24 h. Data are expressed as mean ± S.E.M. of at least 3 independent experiments.

Since PGE_2 _is produced mainly by COX-2 and mPGES-1 in stimulated cells, we investigated whether the inhibitory effect of resveratrol on PGE_2 _was due to regulation of the synthesis of these two enzymes. IL-1β increased the protein synthesis of mPGES-1 and COX-2 (Fig. 2A and 2B). Resveratrol, at doses that significantly inhibited PGE_2 _production, did not significantly reduce mPGES-1 and COX-2 immunoreactivities (Fig. 2A and 2B). In addition, COX-1 protein also remained unaffected (Fig. 2A).

**Figure 3:**
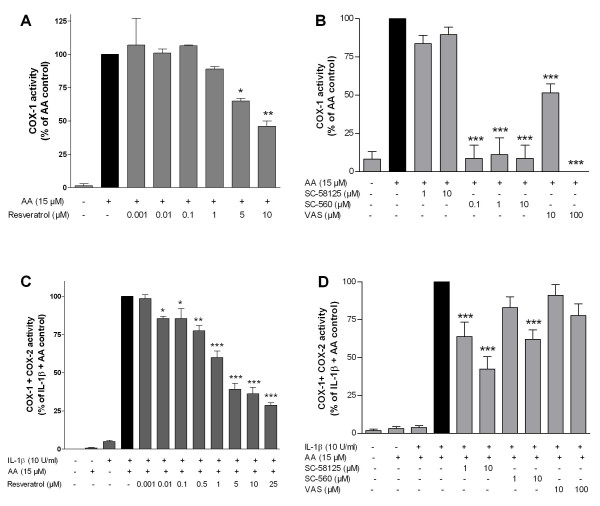
**Effect of resveratrol on COX-1 (A) and total COX (C) activity in SK-N-SH cells**. The arachidonic acid assay was performed as described in the text. Selective inhibitors of COX-1 (SC-560 and VAS) and COX-2 (SC-58125) were included in the COX-1 (**B**) and total COX (**D**) activity assays to assess the relative contribution of each isozyme to the overall COX enzymatic activity. Data are expressed as mean ± S.E.M. of at least 3 independent experiments. *p < 0.05, **p < 0.01 and ***p < 0.001 with respect to IL-1β control (One-way ANOVA followed by the Student-Newman-Keuls *post-hoc *test).

To further clarify the mechanisms responsible for the reduction of PGE_2 _and PGD_2_, we investigated whether resveratrol reduces COX activity. Although resveratrol is a well-known COX-1 inhibitor, it did not inhibit the COX-1 activity at the same doses that reduced PGE_2 _production in SK-N-SH cells (Fig. 3A). A similar effect was obtained with SC-58125 (COX-2 inhibitor), which did not reduce the PGE_2 _production in this assay (Fig. 3B). On the other hand, SC-560 and VAS (COX-1 inhibitors), strongly reduced PGE_2 _formation (Fig. 3B).

Although low concentrations of resveratrol did not inhibit COX-1 activity (Fig. 3A), it significantly reduced total COX activity even at low doses (0.01-5 μM) as shown in an assay used to test both COX-1 and COX-2 activities (Fig. 3C). A strong reduction of PGE_2 _was also observed with SC-58125, a COX-2 inhibitor. However, only a partial reduction in PGE_2 _content was observed with a high concentration of SC-560 (10 μM) and no reduction was observed with VAS, both COX-1 inhibitors (Fig. 3D), confirming our previous results [15]. It is known that the IC_50 _values for SC-560 are 9 nM and 6.3 μM for COX-1 and COX-2, respectively [16]. Therefore, although COX-1 might play a role in this assay, it is possible that at the higher concentration (10 μM), SC-560 also partially inhibits PGE_2 _production by interfering with COX-2 activity. Since at low doses resveratrol did not reduce COX-1 activity (Fig. 3A), inhibition of COX-2 might account for the significant reduction of total COX activity seen at low doses of resveratrol (Fig. 3B).

In the present study, we show that resveratrol, a flavonoid present in some plants and in the red wine, reduced prostanoid synthesis and free radical generation without affecting the expression of COX-2 and mPGES-1 in neuronal cells. This effect may be attributed to the inhibition of COX activity, mainly COX-2, and antioxidant properties.

The search for compounds that have a potential to reduce neuroinflammation has increased exponentially. Resveratrol is in focus for this purpose, because of its plethora of pharmacological effects and since there are evidences suggesting that its metabolites can cross the blood-brain barrier [17-20], although this availability might not be achieved by a dietary administration [21].

The pharmacological properties of resveratrol are of a great interest, since it goes beyond its antioxidant properties. Resveratrol has been shown to reduce the synthesis of iNOS [22] and cytokines [23-25] in different cells. However, just a few papers showed the direct effects of resveratrol on neuronal cells [26-29]. SK-N-SH cells, because of their similarity with primary neuronal cells, are a useful cellular model for studying neuropathological processes that might play a role in neurodegenerative disorders.

Here we demonstrated that resveratrol strongly reduced prostaglandin synthesis without affecting COX-2 and mPGES-1 expression. Recently, using six different human uterine cell lines, Sexton et al. [30] showed that resveratrol, although having opposite effects on COX-2 expression depending on the cell line, reduced PGE_2 _in almost all cell types tested. On the other hand, resveratrol reduced COX-2 promoter activity and expression and PGE_2 _production in different cells [31-34]. We also have recently demonstrated that resveratrol reduces mPGES-1 expression, PGE_2 _and 8-*iso*-PGF_2α _generation, without interfering with COX-2 expression in LPS-stimulated microglia [14]. Therefore, the effect of resveratrol on COX-2 and mPGES-1 expression might be dependent on the cell type.

Similar to our results, resveratrol has been shown to inhibit PGD_2 _production induced by IgE in bone marrow-derived mouse mast cells in vitro [35] and PGD_2 _and COX-2 expression in the colon in a rat model of chronic colitis [36].

Although resveratrol is known as a COX-1 inhibitor, it did not reduce COX-1 activity at the doses that PGE_2 _and PGD_2 _content were reduced. However, COX-2 activity was reduced even at low concentrations that might be important to the reduction of the prostanoids. Other studies have also demonstrated that resveratrol is able to reduce COX-2 activity [14,33,37,38]. Although in our experimental set up resveratrol reduced PG formation in the AA assay probably by inhibiting COX-2 activity, it is possible that it reduced the activity of other PGE synthases, such as mPGES-1, even considering that the experimental conditions used were not appropriated to measure mPGES-1 activity, which demands glutathione and other reagents [39-41]. Moreover, a reduction in the phospholipase A_2 _activity and AA release might also contribute to the reduction in the prostanoid formation [42].

Resveratrol also diminished isoprostane formation in IL-1β-stimulated SK-N-SH cells. This ability can be explained by its antioxidant activity and by the reduction of COX-2 activity induced by resveratrol. We and others have demonstrated that 8-*iso*-PGF_2α _formation can also be dependent on COX-2 activity [13,15,43].

Recently, Jin et al. [44] demonstrated that chronic *per os *administration of resveratrol significantly improved the neurobehavioral deficit and reduced the expression of COX-2 in the substantia nigra in rats administered with 6-hydroxydopamine, a substance used to simulate an animal model of Parkinson's disease. Since the COX-2 levels were measured in a brain region, it is not possible to establish its major cellular source, which could be neurons or glial cells. Resveratrol also reduced the apoptotic dopaminergic neuronal death induced by microglia activation in a microglial-neuronal coculture [29]. The neuroprotection conferred by resveratrol might be related to the ability to reduce the production of prostaglandins and free radicals in neurons and microglia.

In summary, we conclude that resveratrol reduced the prostaglandin formation in neuronal cells probably by inhibiting the activity of inducible PG synthesizing enzymes, such as COX-2, but with a weak influence on COX-1 activity and not interfering with the expression of COX-1, COX-2 or mPGES-1. These effects further support the interest of resveratrol as a potential tool in the treatment of neuroinflammatory conditions.

## Abbreviations

ANOVA: analysis of variance; BSA: bovine serum albumin; COX: cyclooxygenase; DMEM: Dulbecco's modified Eagle's medium; EIA: enzyme immunoassay; iNOS: inducible nitric oxide synthase; IL-1β: interleukin-1β; 8-*iso-*PGF_2α_: 8-*iso*-prostaglandin F_2α_; LPS: lipopolysaccharide; mPGES-1: microsomal prostaglandin E synthase 1; PBS: phosphate-buffered saline; PG: prostaglandin; PVDF: polyvinylidene fluoride; SDS: sodium dodecyl sulfate; TBS: Tris-buffered saline; TBST: Tris-buffered saline containing 0.1% Tween 20.

## Competing interests

The authors declare that they have no competing interests.

## Authors' contributions

ACPO directed the work, contributed to design the study, reviewed the data and wrote the manuscript; LW performed Western blot analysis, COX activity assay and prostaglandin measurements; HSB helped in performing prostaglandin measurements and reviewed the manuscript; ECJ provided consultation and reviewed the data and the manuscript; BLF designed the study, reviewed the data and the manuscript. All authors read and approved the final manuscript.
